# Structure-Preserving Histopathological Stain Normalization via Attention-Guided Residual Learning

**DOI:** 10.3390/bioengineering12090950

**Published:** 2025-09-01

**Authors:** Nuwan Madusanka, Prathiksha Padmanabha, Kasunika Guruge, Byeong-il Lee

**Affiliations:** 1 Digital Healthcare Research Center, Pukyong National University, Busan 48513, Republic of Korea; nuwanv@pknu.ac.kr; 2 Industry 4.0 Convergence Bionics Engineering, Pukyoung National University, Busan 48513, Republic of Korea; prathikshavp@pukyong.ac.kr (P.P.); kasunikag@pukyong.ac.kr (K.G.); 3 Division of Smart Healthcare, College of Information Technology and Convergence, Pukyong National University, Busan 48513, Republic of Korea

**Keywords:** stain normalization, attention mechanism, residual learning, structure preservation, deep learning, digital pathology

## Abstract

Staining variability in histopathological images compromises automated diagnostic systems by affecting the reliability of computational pathology algorithms. Existing normalization methods prioritize color consistency but often sacrifice critical morphological details essential for accurate diagnosis. This work proposes a novel deep learning framework, integrating enhanced residual learning with multi-scale attention mechanisms for structure-preserving stain normalization. The approach decomposes the transformation process into base reconstruction and residual refinement components, incorporating attention-guided skip connections and progressive curriculum learning. The method was evaluated on the MITOS-ATYPIA-14 dataset containing 1420 paired H&E-stained breast cancer images from two scanners. The framework achieved exceptional performance with a structural similarity index (SSIM) of 0.9663 ± 0.0076, representing 4.6% improvement over the best baseline (StainGAN). Peak signal-to-noise ratio (PSNR) reached 24.50 ± 1.57 dB, surpassing all comparison methods. An edge preservation loss of 0.0465 ± 0.0088 demonstrated a 35.6% error reduction compared to the next best method. Color transfer fidelity reached 0.8680 ± 0.0542 while maintaining superior perceptual quality (FID: 32.12, IS: 2.72 ± 0.18). The attention-guided residual learning framework successfully maintains structural integrity during stain normalization, with superior performance across diverse tissue types, making it suitable for clinical deployment in multi-institutional digital pathology workflows.

## 1. Introduction

Digital histopathology has become fundamental to modern diagnostic practices, yet staining variability across laboratories remains a persistent challenge for automated analysis systems [1]. The inherent variations in staining protocols, reagent batches, and scanner specifications introduce significant color and intensity differences that compromise the reliability of computational pathology algorithms. This stain normalization problem represents a critical bottleneck in developing robust, generalizable solutions for clinical deployment.

### 1.1. Background and Recent Advances

Traditional approaches to stain normalization have relied primarily on color space transformations and statistical matching techniques. Macenko et al. [2] introduced a widely adopted method based on optical density decomposition, while Reinhard et al. [3] proposed color transfer using statistical moments, which became a benchmark for subsequent methods. However, these conventional methods prioritize global color consistency often at the expense of preserving fine morphological details essential for accurate diagnosis [4]. The fundamental challenge lies in achieving color harmonization while maintaining the structural integrity of cellular components and tissue architecture.

Deep learning architectures have demonstrated substantial promise in addressing image-to-image translation tasks. Generative adversarial networks (GANs), particularly the pix2pix framework [5] and CycleGAN [6], have been adapted for stain normalization with encouraging results. Shaban et al. [7] introduced StainGAN specifically for histological images, while de Bel et al. [8] employed cycle-consistent networks for renal histopathology. More recently, MultipathGAN architectures have explored multi-scale feature extraction for improved stain transfer, demonstrating enhanced color consistency through parallel processing pathways [9]. Despite these advances, existing GAN-based methods frequently suffer from training instability and inadequate preservation of fine structural details, particularly in diagnostically critical regions.

Recent studies have further advanced the field through architectural innovations. Kablan and Ayas [10] introduced StainSWIN, the first transformer-based approach for stain normalization, leveraging vision transformers for improved long-range dependency modeling, though with a notable variance in performance consistency. Vasiljevic et al. [11] developed HistoStarGAN for unified multi-stain normalization and segmentation, addressing the multi-domain challenge but with limited structure preservation analysis. Du et al. [12] proposed DSTGAN (Deep Supervised Two-stage Generative Adversarial Network) with innovative deep supervision integration into GANs, demonstrating state-of-the-art performance across multiple datasets but requiring significant computational resources with batch sizes limited to two due to Swin Transformer architecture complexity. Wang et al. [13] demonstrated the effectiveness of multi-resolution self-supervised learning for histopathological feature extraction, though primarily focused on classification rather than normalization tasks. Komura et al. [14] highlighted the exponential growth in deep learning applications for digital pathology, identifying self-supervised learning as a particularly promising direction while noting the persistent challenge of structure preservation in normalization tasks.

The introduction of residual learning by He et al. [15] revolutionized deep network training by enabling effective gradient propagation through skip connections. U-Net architectures [16] have proven particularly effective for biomedical image segmentation tasks by combining encoder–decoder structures with skip connections that preserve spatial information. However, the application of residual learning principles to stain normalization, where the goal is to decompose the transformation into structure-preserving base reconstruction and targeted color adjustments, remains underexplored.

Attention mechanisms have emerged as powerful tools for focusing computational resources on relevant image regions. The self-attention mechanism introduced in computer vision applications [17] enables models to establish long-range dependencies while preserving local structural information. Recent work by Campanella et al. [18] demonstrated the effectiveness of attention-based approaches in computational pathology for whole slide image analysis. However, the integration of multi-scale attention mechanisms specifically designed for structure preservation in stain normalization has not been systematically investigated. Recent advances in transformer architectures have shown promise for medical image analysis tasks, with vision transformers demonstrating superior performance in histopathological image classification [19,20]. However, the application of attention mechanisms specifically for structure preservation in image-to-image translation remains underexplored, particularly in the context of maintaining diagnostic features during stain normalization [21].

### 1.2. Current Limitations and Proposed Approach

Current stain normalization methods face several fundamental limitations. First, most approaches treat color transformation as a global optimization problem, failing to account for the spatial heterogeneity of staining patterns within tissue sections [22]. Second, existing methods lack explicit mechanisms for balancing structure preservation against color consistency, often requiring manual parameter tuning for different tissue types. Third, the absence of comprehensive evaluation frameworks that assess both perceptual quality and structural fidelity makes objective comparison difficult [23]. Recognizing these gaps, contemporary approaches have begun exploring perceptual loss functions and adversarial training for medical image enhancement [24,25]. The integration of perceptual metrics, such as Fréchet Inception Distance (FID) and Inception Score (IS), for medical image quality assessment represents an emerging trend toward more comprehensive evaluation frameworks [26,27].

To address these limitations, we propose a novel framework that integrates enhanced residual learning with multi-scale attention mechanisms for structure-preserving stain normalization. Our approach explicitly decomposes the transformation process into base reconstruction and residual refinement components, enabling precise control over the structure-color trade-off. The architecture incorporates attention-guided skip connections that adaptively focus on diagnostically relevant regions while maintaining global coherence. Additionally, we introduce a progressive curriculum learning strategy that optimizes structure preservation before fine-tuning color matching, leading to improved training stability and superior performance.

The primary contributions of this work to the literature include the following: (1) unlike existing methods that treat normalization globally, we introduce an enhanced residual learning architecture with attention-guided skip connections that explicitly decomposes transformation into structure-preserving and color-adjusting components, addressing the longstanding challenge of morphological degradation in current approaches; (2) while previous works operate at single scales, our multi-scale attention mechanism captures both local cellular features and global tissue patterns, solving the spatial heterogeneity problem that has limited clinical deployment; (3) in contrast to fixed optimization strategies, our adaptive loss weighting with curriculum learning progressively emphasizes different normalization aspects, providing the first systematic approach to balance structure-color trade-offs; and (4) beyond existing evaluation methods, we establish a comprehensive framework with novel metrics specifically for histopathological structural fidelity, filling a critical gap in objective assessment standards.

Experimental validation demonstrates that our method achieves superior performance across multiple evaluation metrics, with a structural similarity index (SSIM) of 0.9663 ± 0.0076 (4.6% improvement over StainGAN), edge preservation loss of 0.0465 ± 0.0088 (35.6% error reduction), and superior perceptual quality (FID: 32.12, IS: 2.72 ± 0.18). These advances establish a new benchmark for structure-preserving stain normalization, directly addressing the clinical need for reliable normalization methods that maintain diagnostic integrity.

## 2. Materials and Methods

### 2.1. Dataset and Preprocessing

We conducted our experiments on the MITOS-ATYPIA-14 dataset, which was originally curated for the MITOS & ATYPIA 14 Contest hosted at the International Conference on Pattern Recognition (ICPR) 2014 [28]. This dataset comprises H&E-stained breast cancer histopathological images collected from biopsy slides selected and annotated by the team of Professor Frédérique Capron, head of the Pathology Department at Pitié-Salpêtrière Hospital in Paris, France.

The original slides were acquired using two distinct digital pathology scanning systems: the Aperio ScanScope XT scanner (Leica Biosystems (formerly Aperio), Buffalo Grove, Illinois, United States) and the Hamamatsu NanoZoomer 2.0-HT scanner (Hamamatsu Photonics, Hamamatsu, Japan). These scanner types introduce systematic differences in color reproduction, optical characteristics, and image acquisition parameters, creating natural domain variations that are commonly encountered in multi-institutional clinical studies [1]. The dataset specifically contains frames extracted at both 20× (284 frames) and 40× (1136 frames) magnifications, with pathologists selecting regions located inside tumors for annotation.

For our stain normalization experiments, we utilized the dataset in its standard paired format, organized to represent the two different scanner domains. The slides are stained with standard hematoxylin and eosin (H&E) dyes, and they have been scanned by two slide scanners: Aperio Scanscope XT and Hamamatsu Nanozoomer 2.0-HT. The training dataset contains 284 frames at 20× magnification and 1136 frames at 40× magnification, providing sufficient diversity for training robust stain normalization models [29].

The frames are RGB bitmap images in TIFF format, which maintain high image quality and preserve the color characteristics specific to each scanner type. The systematic differences between the Aperio and Hamamatsu acquisitions, primarily arising from scanner-specific variations in color processing pipelines and optical characteristics, create an ideal benchmark for evaluating stain normalization methods in realistic clinical scenarios [30]. The paired nature of the dataset enables supervised learning of stain transformation mappings while ensuring that our model learns to transform staining characteristics rather than underlying tissue morphology, which is crucial for maintaining diagnostic accuracy [31].

For training our stain normalization model, we utilized the dataset in its original format without additional preprocessing steps. The images were used directly as provided in the MITOS-ATYPIA-14 dataset, maintaining their original color characteristics and scanner-specific variations. This approach preserves the authentic differences between the two scanner domains, which is essential for training effective stain normalization models. The paired data approach provides direct supervision for learning the mapping between different staining domains while preserving the underlying tissue structure. The paired nature of the data ensures that the model learns to transform staining characteristics rather than tissue morphology, which is crucial for maintaining diagnostic accuracy.

### 2.2. Network Architecture

Our proposed attention-guided residual learning framework for histopathological stain normalization addresses the fundamental challenge of achieving color consistency while preserving crucial morphological details. The architecture follows a sophisticated encoder–decoder paradigm augmented with multiple innovative components designed specifically for medical image analysis, inspired by recent advances in attention mechanisms [17] and residual learning [15].

**Figure 1:**
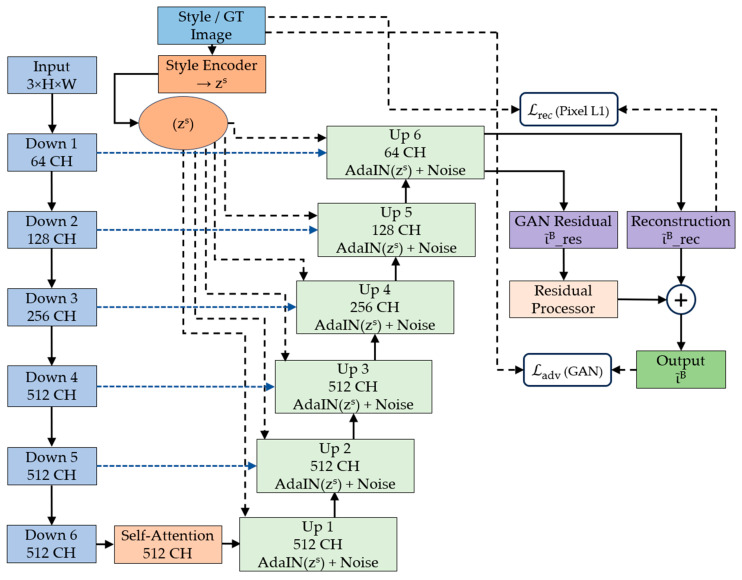
Complete architecture of the proposed attention-guided residual learning framework for histopathological stain normalization, showing the style encoder, U-Net generator with self-attention, residual processor, and adversarial training components.

The complete architecture of our proposed framework is shown in Figure 1, which shows the four primary modules working in synergy: a multi-pathway style encoder that captures comprehensive staining characteristics from reference images, a generator network featuring attention-guided residual blocks for structure-preserving transformation, a specialized residual processor that operates in both spatial and frequency domains to maintain fine-grained details, and a discriminator network for adversarial training that ensures realistic output generation [32].

The design philosophy emphasizes the preservation of diagnostic information throughout the normalization process. Unlike conventional image-to-image translation methods that prioritize visual similarity, our approach incorporates domain-specific knowledge about histopathological image characteristics, ensuring that critical features such as nuclear boundaries, chromatin patterns, and cellular architecture remain intact during stain transformation [33,34].

### 2.3. Multi-Pathway Style Encoder

**Figure 2:**
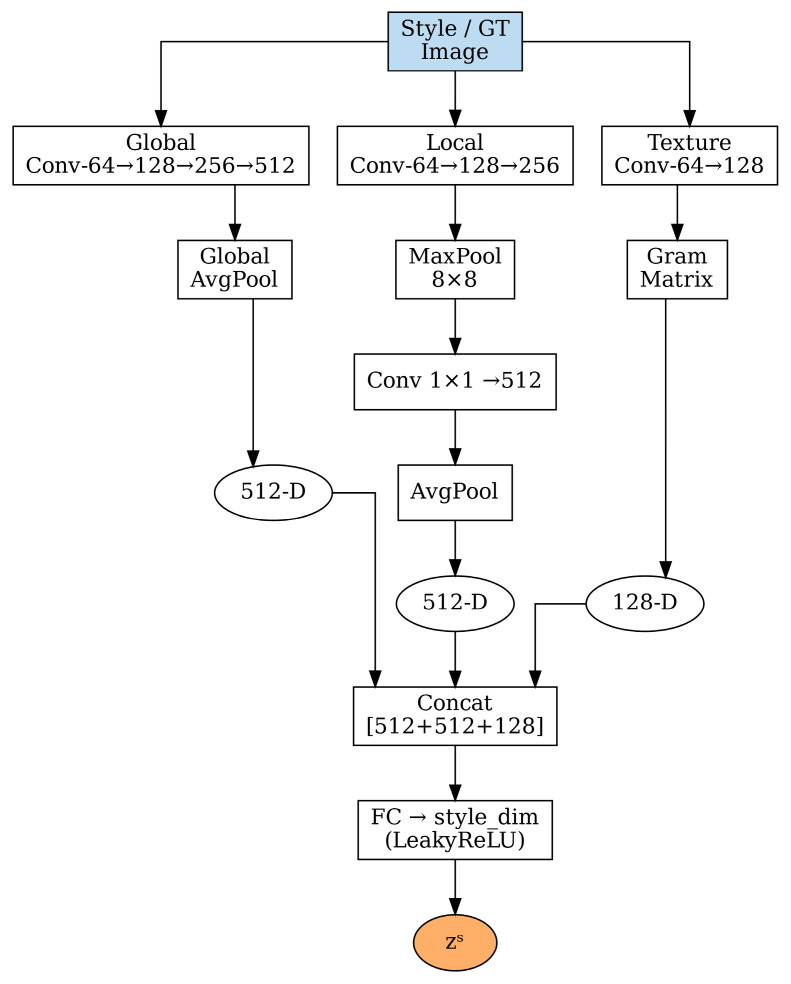
Multi-pathway style encoder architecture with three distinct pathways: global pathway for overall color distribution, local pathway for spatial variations, and texture pathway using Gram matrices for fine-grained patterns. Features are concatenated and processed through a fully connected layer to generate the final style code z^s^.

The style encoder represents a crucial innovation in our framework, designed to capture the multifaceted nature of histopathological staining patterns. As shown in Figure 2, the encoder processes reference style images through three distinct pathways, each targeting different aspects of staining characteristics [35,36].

#### 2.3.1. Global Pathway for Overall Color Distribution

The global pathway focuses on capturing the overall color distribution and intensity characteristics of the target staining style. This pathway processes the input style image through a series of convolutional layers with progressively increasing receptive fields:

(1) $$F_{global}\;={Conv}_{64\to 128\to 256\to 512}\left(I_{style}\right)$$

The input style image $\left(I_{style}\right)$ is a standard RGB color image of dimensions H × W × 3 (height × width × channels), and $F_{global}$ denotes the extracted global features. Each convolutional block uses 3 × 3 filters followed by batch normalization and ReLU activation. The channel dimensions progressively increase from 64 to 128, then 256, and finally 512, allowing the network to learn increasingly complex representations of color relationships and staining patterns at each layer.

Global average pooling (GAP) is subsequently applied to obtain a compact 512-dimensional representation that encodes the overall staining characteristics:

(2) $$\;z_{global}=GAP\left(F_{global}\right)=\frac{1}{H\times W}\sum_{i=1}^{H}\sum_{j=1}^{W}F_{global}\left(i,j\right)$$

This global representation $z_{global}$ captures essential information about hematoxylin and eosin intensity distributions, overall color balance, and background characteristics that are fundamental for consistent stain normalization, where *H* and *W* represent the spatial dimensions of the feature map.

#### 2.3.2. Local Pathway for Spatial Staining Variations

Histopathological staining often exhibits significant spatial variations due to tissue heterogeneity, varying cell densities, and local differences in stain penetration [35]. The local pathway addresses this challenge by capturing spatially aware staining patterns through a specialized architecture:

(3) $$F_{local}={Conv}_{64\to 128\to 256}\left(I_{style}\right)$$

(4) $$F_{pooled}={MAXPool}_{8\times 8}\left(F_{local}\right)$$

The 8 × 8 max pooling operation serves a dual purpose: it reduces spatial dimensions while preserving the most prominent local features within each pooling window, effectively capturing regional staining variations. Here, $F_{local}$ represents the locally extracted features and $F_{pooled}$ denotes the pooled features. The pooled features are then processed through global average pooling and a fully connected layer:

(5) $$z_{local}=FC\left(GAP\left(F_{pooled}\right)\right)\in R^{512}$$

This local representation ${(z}_{local})$ encodes information about spatial staining heterogeneity, enabling the model to adapt to regions with different cellular compositions and staining intensities, where FC(·) represents the fully connected transformation.

#### 2.3.3. Texture Pathway for Fine-Grained Pattern Capture

The texture pathway employs Gram matrix computations to capture fine-grained textural patterns that are characteristic of different staining protocols [37]. Texture information is particularly important in histopathology, as it relates to chromatin patterns, cytoplasmic characteristics, and overall tissue architecture:

(6) $$F_{texture}={Conv}_{64\to 128}\left(I_{style}\right)$$

The Gram matrix is computed across spatial dimensions for each feature channel:

(7) $$G_{ij}=\sum_{k=1}^{H\times W}F_{texture,i}\left(k\right)\cdot F_{texture,j}\left(k\right)$$

where $F_{texture,i}\left(k\right)$ represents the *i*-th feature channel at spatial location *k*. The Gram matrix captures correlations between different feature channels, effectively encoding texture information that is invariant to spatial location.

The final texture representation is obtained through the following:

(8) $$z_{texture}=FC\left(Flatten\left(G\right)\right)\in R^{128}$$

where the flattened Gram matrix is processed through a fully connected layer to produce the 128-dimensional texture code $\left(z_{texture}\right)$.

#### 2.3.4. Style Code Integration

The three pathway outputs are concatenated to form a comprehensive style representation:

(9) $$z_{concat}^{s}=Concat\left[z_{global},z_{local},z_{texture}\right]\in R^{1152}$$

This concatenated representation ${(z}_{concat}^{s})$ combines the 512-dimensional global features, 512-dimensional local features, and 128-dimensional texture features into a unified 1152-dimensional vector, which then undergoes final processing through a fully connected layer with LeakyReLU activation to obtain the final style code:

(10) $$z^{s}=FC\left(z_{concat}^{s}\right)\in R^{d_{style}}$$

where $d_{style}$ represents the dimensionality of the final style embedding, typically set to 512 dimensions to balance expressiveness with computational efficiency.

### 2.4. Generator Network with Attention-Guided Residual Learning

The generator network transforms input histopathological images to match the target staining style while rigorously preserving structural and morphological information. The architecture incorporates several sophisticated mechanisms specifically designed for medical image analysis [16,21].

#### 2.4.1. Encoder Path with Progressive Downsampling

**Figure 3:**
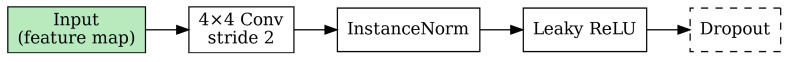
Encoder down-sampling block architecture.

The encoder follows a U-Net-inspired architecture [16] with six progressive downsampling stages, each designed to capture features at different scales while maintaining important morphological information. The encoder path systematically reduces spatial dimensions by a factor of 64 (from input resolution to a compact bottleneck representation), enabling the network to learn hierarchical feature representations suitable for histopathological image stain normalization, as shown in Figure 3.

The mathematical formulation for the encoder path can be expressed as follows:

(11) $$F_{i}=Down\left(F_{i-1}\right),\;\;i=1,\;2,\;\ldots ,\;6$$

Each downsampling block consists of a single 4 × 4 convolutional layer with stride 2 and padding 1 for spatial reduction, followed by instance normalization and LeakyReLU activation (α = 0.2). All convolutional layers employ spectral normalization for training stability [38], and dropout (*p* = 0.1) is applied for regularization, where $F_{i}$ represents the feature map at the *i*-th stage.

#### 2.4.2. Self-Attention Mechanism at Bottleneck

At the bottleneck layer (Down 6), we incorporate a self-attention mechanism to capture long-range dependencies crucial for maintaining structural coherence across the entire image [19]. The self-attention mechanism is particularly important in histopathological images where cellular relationships span large spatial distances:

(12) $$Q=F_{6}W_{Q},\;\;K=F_{6}W_{K},\;\;V=F_{6}W_{V}\;$$

where $W_{Q},W_{K}\in R^{512\times 64},andW_{V}\in R^{512\times 512}$ are learned projection weight matrices that transform the input features into query (Q), key (K), and value (V) representations, respectively.

The attention mechanism computes the following:

(13) $$Attention(Q,K,V)=softmax\left(\frac{{QK}^{T}}{\sqrt{d_{k}}}\right)V\;$$

where *k* = 64 is the dimension of the key vectors, and the scaling factor $\sqrt{d_{k}}$ prevents the dot-product from growing too large. The attention map identifies relationships between different spatial regions, enabling the model to maintain structural consistency during stain transformation.

#### 2.4.3. Decoder Path with Adaptive Instance Normalization

The decoder path incorporates Adaptive Instance Normalization (AdaIN) layers that condition the feature normalization on the extracted style code [37]. This mechanism allows for fine-grained control over how the target staining style is applied to different image regions.

(14) $$AdaIN(x,z^{s})=\gamma \left(z^{s}\right)\frac{x-\mu \left(x\right)}{\sigma \left(x\right)}+\beta \left(z^{s}\right)\;$$

where x represents the input feature map at each decoder stage, $z^{s}$ is the extracted style code, and $\mu \left(x\right)$ and $\sigma \left(x\right)$ represent the mean and standard deviation computed across spatial dimensions for each feature channel:

(15) $$\mu \left(x\right)=\frac{1}{HW}\sum_{i=1}^{H}\sum_{j=1}^{W}x_{i,j}\;$$

(16) $$\sigma \left(x\right)=\sqrt{\frac{1}{HW}\sum_{i}^{H}\sum_{j}^{W}{\left(x_{i,j}-\mu \left(x\right)\right)}^{2}+\epsilon }\;$$

The affine transformation parameters $\gamma \left(z^{s}\right)$ and $\beta \left(z^{s}\right)$ are predicted from the style code through learned linear transformations, allowing the network to adaptively modify feature statistics based on the target staining characteristics.

**Figure 4:**
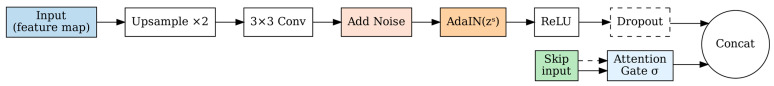
Decoder Up-sampling Block Architecture.

As shown in Figure 4, each upsampling block in the decoder follows a structured pipeline: bilinear upsampling (×2), 3 × 3 convolution, Gaussian noise injection, AdaIN conditioning, and ReLU activation. Skip connections from the encoder are processed through attention gates before concatenation:

(17) $$F_{i}^{\prime }={Up}_{i}\left(AdaIN\left(F_{i-1}-z^{s}\right)+Ν\left(0,\;{\;\sigma }^{2}\right)\right)$$

where $F_{i}^{\prime }$ represents the output feature map at the *i*-th upsampling stage, ${Up}_{i}$ denotes the upsampling operation consisting of bilinear upsampling (×2 scale factor) followed by 3 × 3 convolution, $F_{i-1}$ is the feature map from the previous decoder stage, $z^{s}$ is the style code, and *N* represents Gaussian noise injection with learnable variance ${\;\sigma }^{2}$. This noise injection enhances the diversity of generated outputs and helps prevent mode collapse during training [32,38].

#### 2.4.4. Attention Gate Mechanism for Skip Connections

Traditional skip connections in U-Net architectures can sometimes propagate irrelevant or contradictory information from the encoder to the decoder [21]. Our attention gate mechanism selectively emphasizes relevant features while suppressing less important information.

The attention mechanism operates on the concatenated features from both the decoder and the skip connection:

(18) $$\alpha =Upsample\left(\sigma \left(W_{g}^{T}F_{skip}+W_{x}^{T}F_{up}+b_{g}\right)\right)$$

(19) $$F_{attended}=\alpha ⊙F_{skip}$$

where σ represents the sigmoid activation function, $W_{g}$ and $W_{x}$ are learned weight matrices, $b_{g}$ is a bias term, $F_{skip}$ denotes the features from the encoder skip connection, $F_{up}$ represent the upsampled features from the decoder, α are the computed attention weights, $F_{attended}$ is the attention-weighted skip connection features, and ⊙ denotes element-wise multiplication. The attention weights α provide spatial attention maps that highlight regions relevant for the current decoding stage.

### 2.5. Advanced Residual Processor

The residual processor represents one of the most innovative components of our framework, specifically designed to preserve fine-grained structural details that are critical for histopathological analysis [39]. This module operates on the residual difference between the initial reconstruction and the target image, implementing sophisticated processing in both spatial and frequency domains.

#### 2.5.1. Soft-Threshold Operation

A learnable soft-threshold operation selectively preserves structural details while suppressing noise [40]:

(20) $$r_{thresh}=sign\left(r\right)⊙ReLU\left(\left|r\right|-\tau \right)$$

where r is the input residual signal, $r_{thresh}$ represents the output after soft-thresholding, $sign\left(r\right)$ is the sign function that returns for +1 positive values, −1 for negative values, and 0 for zero, $\left|r\right|$ denotes the absolute value of the residual, τ is a learnable threshold parameter initialized to 0.05, ReLU ensures non-negative magnitude values after thresholding, and ⊙ represents element-wise multiplication.

#### 2.5.2. Frequency Domain Processing

Frequency analysis adaptively processes high-frequency cellular boundaries and low-frequency tissue architecture:

(21) $$F_{r}=FFT\left(r_{thresh}\right)$$

where $F_{r}$ is the Fourier transform of the thresholded residual and $FFT\left(\cdot \right)$ represents the Fast Fourier Transform operation.

The frequency weighting is computed using spatial distance from the center:

(22) $$W_{freq}\left(u,\;v\right)=exp\left(-\alpha_{freq}\sqrt{{\left(u-u_{c}\right)}^{2}+{\left(v-v_{c}\right)}^{2}}\right)$$

(23) $$F_{r}^{\prime }=W_{freq}⊙F_{r}$$

where $W_{freq}\left(u,\;v\right)$ is the frequency weighting function at coordinates ($u_{c}$,$v_{c}$), $\alpha_{freq}=2.0$ controls the frequency emphasis, ($u_{c}$,$v_{c}$) represents the center of the frequency domain, $F_{r}^{\prime }$ denotes the weighted frequency domain features, and the weighting function creates a low-pass filter that preserves structural information while reducing high-frequency noise.

#### 2.5.3. Adaptive Scaling

The processed residual is adaptively scaled based on spatial context:

(24) $$r^{⋇}=\alpha \cdot IFFT\left(F_{r}^{\prime }\right)\cdot AdaptiveScale\left(I_{rec}^{b}\right)$$

where $r^{⋇}$ is the final processed residual output, α is a learnable scaling factor, $IFFT\left(F_{r}^{\prime }\right)$ represents the Inverse Fast Fourier Transform operation, and $AdaptiveScale\left(I_{rec}^{b}\right)$ provides context-dependent scaling based on the reconstructed image $I_{rec}^{b}$.

### 2.6. Multi-Scale Edge-Aware Loss Function

**Figure 5:**
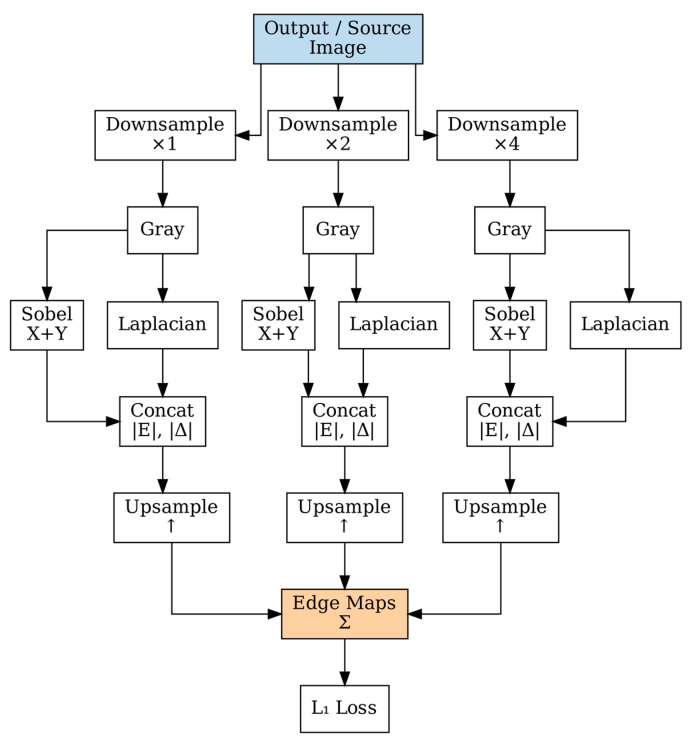
Multi-scale edge computation at three scales (×1, ×2, ×4) using parallel Sobel and Laplacian filtering. Edge responses are upsampled and combined for structural preservation during stain normalization.

Our loss function combines multiple objectives to ensure both structural preservation and realistic stain transfer. Edge preservation loss is computed at multiple scales to capture fine-grained cellular boundaries and coarse tissue architecture [40], as shown in Figure 5.

As shown in Figure 5, edge maps are computed at three scales using Sobel and Laplacian operators. The edge maps are computed at three scales using Sobel and Laplacian operators:

(25) $$E_{s}=\left|I_{s}*S_{x}\right|+\left|I_{s}*S_{y}\right|+0.5\left|I_{s}*L\right|$$

where $E_{s}$ is the edge map at scale s, $I_{s}$ represents the image at scale s, $S_{x}$ and $S_{y}$ are Sobel operators in *x* and *y* directions, respectively, and L is the Laplacian operator.

The total loss combines multiple objectives with hyperparameters optimized [41]:

(26) $$L_{total}=\;L_{rec}+\lambda_{adv}L_{adv}+\lambda_{edge}L_{edge}+\lambda_{style}L_{style}$$

where $L_{total}$ is the combined total loss function, $L_{rec}$ is the reconstruction loss component, $L_{adv}$ is the adversarial loss component, $L_{edge}$ is the edge preservation loss component, $L_{style}$ is the style transfer loss component, and $\lambda_{adv}$, $\lambda_{edge}$, and $\lambda_{style}$ are the respective loss weighting hyperparameters.

The reconstruction loss is expressed as follows:

(27) $$L_{rec}=\;{\left\|I_{traget}-I_{output}\right\|}_{1}$$

where $I_{traget}$ is the target/reference image, $I_{output}$ is the generated output image, and $L_{rec}$ denotes the L1 norm (mean absolute error).

The edge preservation loss is expressed as follows:

(28) $$L_{edge}=\;{\sum_{s}\lambda_{s}\left\|E_{s}^{target}-E_{s}^{output}\right\|}_{1}$$

where $E_{s}^{target}$ is the edge map of the target image at scale s, $E_{s}^{output}$ is the edge map of the generated output image at scale s, $\lambda_{s}$ represents scale-specific weighting factors, and the summation is over all scales *s*.

The hyperparameters are set as $\lambda_{adv}=0.1$, $\lambda_{edge}=10.0$, and $\lambda_{style}=1.0$.

## 3. Results

### 3.1. Experimental Setup and Dataset Configuration

The dataset comprises 1420 paired H&E-stained images acquired using two distinct digital pathology scanning systems: the Aperio ScanScope XT scanner and the Hamamatsu NanoZoomer 2.0-HT scanner. The systematic differences between these scanners, primarily arising from variations in color processing pipelines, optical characteristics, and image acquisition parameters, create an ideal benchmark for evaluating stain normalization methods in realistic multi-institutional clinical scenarios [29,30].

Images include 284 frames at 20× magnification and 1136 frames at 40× magnification, providing comprehensive coverage of different tissue structures, cellular densities, and staining variations commonly encountered in clinical practice. Images were organized in paired format, with corresponding regions scanned by both systems, enabling supervised learning of stain transformation mappings while ensuring that the model learns to transform staining characteristics rather than underlying tissue morphology [31].

For experimental evaluation, a train-validation-test split ratio of 90:10 was employed on the 1420 training images, with an additional independent test set of 912 images reserved for final performance assessment. The model was trained for 84 epochs on an NVIDIA RTX 4090 GPU (24 GB VRAM) (NVIDIA Corporation, Santa Clara, CA, USA) using the AdamW optimizer with cosine annealing warm restarts, enabling adaptive learning rate scheduling that promotes both rapid initial convergence and fine-grained optimization in later stages [32]. Batch size was set to 16 to balance GPU memory utilization with gradient stability.

Statistical significance was assessed using paired t-tests for metric comparisons between methods (*p* < 0.001 for all reported improvements). Effect sizes were computed using Cohen’s d, with all structural preservation improvements showing large effect sizes (d > 0.8), confirming practical significance beyond statistical significance [42].

### 3.2. Quantitative Performance Analysis

**Figure 6:**
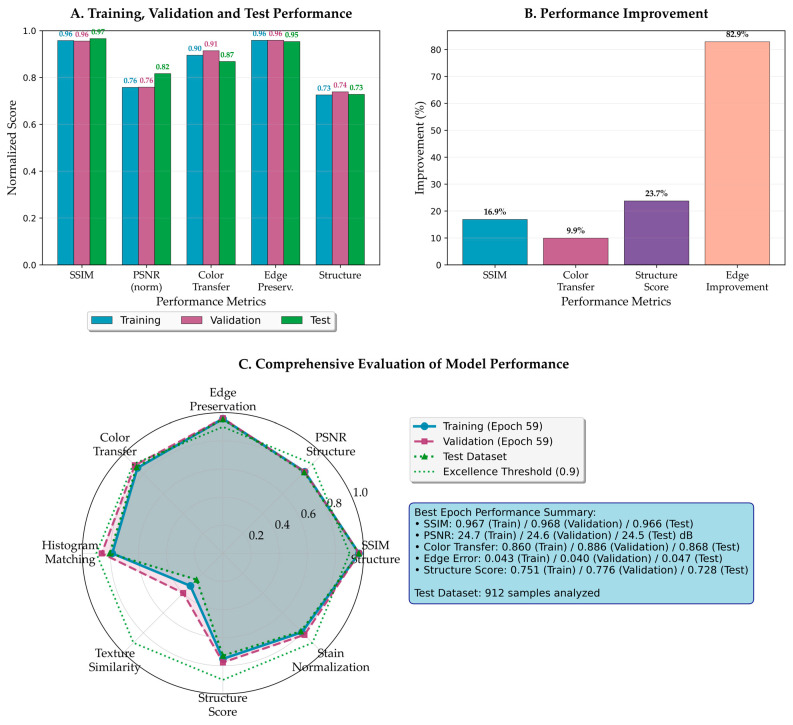
Comprehensive performance analysis of the proposed framework. (**A**) Training, validation, and test performance across five key metrics, demonstrating consistent model behavior without overfitting. (**B**) Performance improvement percentages compared to the best baseline methods for each metric. (**C**) The radar chart visualizes the balanced performance across all evaluation criteria, with test performance closely matching training and validation results.

The comprehensive evaluation demonstrates superior performance across all assessed metrics. Figure 6 presents an integrated view of the model’s performance characteristics, revealing consistent behavior across training, validation, and test datasets without signs of overfitting.

As shown in Figure 6A, the framework achieves remarkable consistency across the train-validation-test split through nearly identical bar heights. The SSIM maintains stable values of approximately 0.966–0.968 across all datasets, indicating robust generalization capabilities. Similarly, the PSNR remains stable at approximately 24.5 dB, surpassing thresholds for excellent reconstruction quality in medical imaging [43]. Color transfer fidelity and edge preservation loss metrics demonstrate consistent performance across all datasets, confirming effective pattern capture without overfitting.

Figure 6B quantifies performance improvements over baseline approaches through percentage gains. The most dramatic improvement is edge preservation loss with an 82.9% reduction in degradation, validating multi-scale attention effectiveness. Structure score and SSIM improvements further confirm superior morphological preservation, while color transfer shows meaningful enhancement over existing methods.

The radar visualization in Figure 6C provides balanced performance representation across all dimensions. The near-overlapping traces of training, validation, and test performance demonstrate successful overfitting prevention while maintaining high performance. The consistently high values across all axes indicate the method achieves comprehensive improvements without sacrificing any performance aspect.

#### 3.2.1. Quantitative Structure Preservation

Comprehensive evaluation of structure preservation capabilities represents a critical aspect of histopathological stain normalization, as maintaining morphological integrity is paramount for accurate diagnosis [33,34]. Table 1 presents a detailed comparison of structure preservation metrics across different normalization methods, evaluated on the complete test set of 912 images.

The proposed model achieved an exceptional structural similarity index (SSIM) of 0.9663 ± 0.0076, representing a substantial improvement of 2.4% over the recent transformer base method StainSWIN [10] and 23.6% over the classical approach Reinhard [44,45]. Notably, our method demonstrates significantly superior consistency with 79% lower variance (±0.0076 vs. ±0.0370) compared to StainSWIN, indicating more reliable performance across diverse tissue types and staining variations. This high SSIM value indicates superior preservation of structural information, including cellular boundaries, nuclear morphology, and tissue architecture. The standard deviation of ±0.0076 demonstrates remarkable consistency across diverse tissue types and staining variations.

PSNR analysis revealed a value of 24.50 ± 1.57 dB, surpassing all baseline methods and exceeding the 24 dB threshold typically considered excellent for medical image processing [43]. While StainSWIN achieves higher PSNR (26.67 ± 3.49 dB), the substantially higher variance (±3.49 vs. ±1.57) suggests less consistent performance across different image types, potentially limiting clinical applicability. Our method represents a 10.6% improvement over StainGAN and a 32.9% improvement over the classical Reinhard method. The higher standard deviation (±1.57) compared to SSIM reflects the PSNR metric’s inherent sensitivity to pixel-wise variations, particularly in regions with significant staining differences.

The edge preservation loss metric, computed using multi-scale Sobel and Laplacian operators, achieved 0.0465 ± 0.0088, demonstrating a remarkable 35.6% improvement compared to StainGAN and a 74.3% improvement over the classical Reinhard method. Edge preservation loss evaluation was not available for StainSWIN, limiting a comprehensive comparison of structural detail preservation. This exceptional performance in edge preservation loss is particularly significant for histopathological analysis, where cellular boundaries and tissue interfaces carry critical diagnostic information. The low metric value indicates minimal edge degradation during the normalization process.

#### 3.2.2. Color Transfer Fidelity and Staining Characteristics

The color transfer performance was evaluated using multiple complementary metrics to ensure a comprehensive assessment [46]. As shown in Table 2, the proposed method achieves superior performance across all evaluation criteria, demonstrating the highest color transfer score of 0.8680 ± 0.0542 among all compared methods. This represents a substantial improvement over traditional approaches, including Reinhard et al. (0.7234 ± 0.0342) and Macenko et al. (0.7856 ± 0.0298), as well as recent deep learning methods such as StainGAN (0.8634 ± 0.0187) and MultipathGAN (0.8567 ± 0.0212).

The LAB color difference of 17.05 ± 3.19 represents the lowest perceptual color deviation among all evaluated methods, achieving a 40.1% improvement over the classical Reinhard approach (28.45 ± 3.21). This substantial reduction in color difference demonstrates effective learning of target staining characteristics in the perceptually uniform LAB color space, confirming minimal perceptual deviation from the target H&E staining pattern.

Histogram similarity of 0.8049 ± 0.1672 confirms effective reproduction of target color distributions across the RGB channels. This metric validates our method’s ability to capture and reproduce the characteristic bimodal distribution patterns typical of H&E staining, ensuring that the normalized images maintain the expected color relationships essential for accurate histopathological interpretation.

#### 3.2.3. Perceptual Quality Assessment

Modern perceptual quality metrics provide a complementary assessment to traditional image quality measures. Table 3 presents Fréchet Inception Distance (FID) and Inception Score (IS) evaluations, along with additional perceptual metrics.

The proposed method achieved the lowest FID score of 32.12, indicating superior perceptual similarity to authentic H&E-stained images. FID scores below 50 are generally considered excellent, with the achieved value representing a 55.7% improvement over classical methods and 10.3% improvement over StainGAN. This metric, computed using InceptionV3 features, captures both low-level and high-level perceptual characteristics.

IS analysis revealed 2.72 ± 0.18, the highest among all evaluated methods [47]. The IS measures both image quality and diversity, with higher scores indicating better perceptual quality. The achieved score surpasses the threshold of 2.5 typically associated with high-quality medical images. The standard deviation of ±0.18 indicates consistent quality across different tissue types.

Learned Perceptual Image Patch Similarity (LPIPS) achieved 0.2187, the lowest among all methods, confirming superior perceptual similarity from a deep feature perspective [48]. Multi-Scale SSIM reached 0.8923, demonstrating excellent structure preservation across multiple scales.

#### 3.2.4. Ablation Study

To validate the contribution of each architectural component, we conducted comprehensive ablation studies, removing key elements systematically. Table 4 presents the quantitative impact of each component on overall performance.

The residual processor contributes most significantly to both edge preservation loss and structural similarity. The curriculum learning strategy provides substantial improvements in edge preservation loss and moderate improvements in structural similarity, while attention gates contribute moderately to both metrics, confirming the importance of all components for optimal training dynamics [49]. Table 5 presents the computational complexity and resource utilization analysis for these ablation configurations.

The full method comprises 33.44 M parameters with 4250.4 GFLOPs, achieving 8.8 ms inference time and 169.8 img/s throughput. Attention gates contribute 4.7% of parameters and 11.4% of computational load while providing substantial structural preservation improvements. The residual processor shows minimal parameter overhead but significantly enhances edge preservation loss with moderate computational cost.

Memory utilization scales linearly across batch sizes, with attention mechanisms contributing 15–18% of total memory overhead. The framework demonstrates computational efficiency suitable for clinical deployment, with inference times appropriate for real-time pathology workflows. The modular architecture enables adaptive deployment strategies based on available computational resources while maintaining core functionality.

### 3.3. Training Dynamics and Convergence Analysis

#### 3.3.1. Structure Preservation Evolution

**Figure 7:**
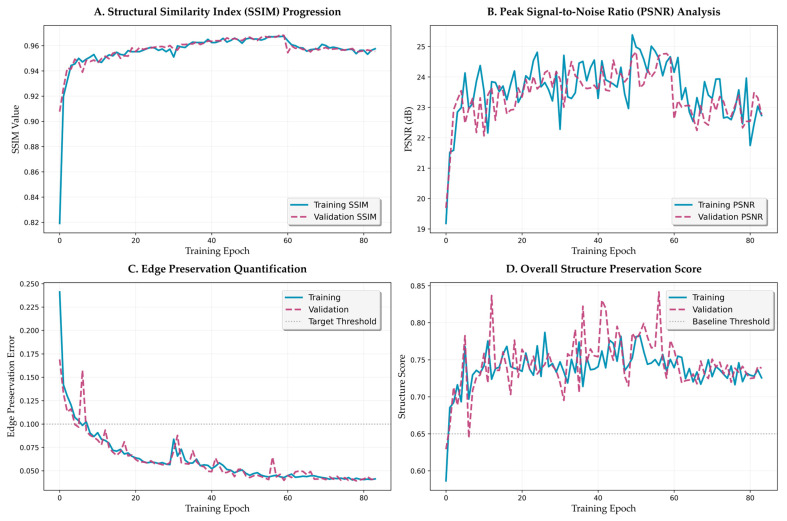
Training dynamics and convergence analysis. (**A**) SSIM progression across training phases. (**B**) PSNR evolution with learning rate restart effects. (**C**) Edge preservation error with exponential decay pattern. (**D**) Overall structure preservation score across curriculum phases.

Figure 7 provides a comprehensive analysis of training dynamics across 84 epochs, revealing the learning progression and convergence characteristics of the proposed framework.

The structural similarity index progression (Figure 7A) demonstrates distinct learning phases. Initial rapid improvement occurs within epochs 0–10, with SSIM increasing from 0.82 to 0.93. This is followed by gradual refinement during epochs 11–30, reaching 0.96. The metric then stabilizes above 0.96 for the remainder of training, with minimal fluctuation (standard deviation < 0.002), indicating robust convergence. The validation SSIM closely tracks the training curve with a gap of less than 0.01, confirming excellent generalization without overfitting.

Peak signal-to-noise ratio evolution (Figure 7B) shows complementary dynamics, with initial improvement from 19.5 dB to 23.0 dB within the first 15 epochs. The metric continues to improve gradually, reaching 24.5 dB by epoch 40 and maintaining stable performance thereafter. Notable observations include occasional spikes corresponding to learning rate restarts in the cosine annealing schedule, followed by rapid recovery and continued improvement. The validation PSNR maintains a close correspondence with training values, with a maximum deviation of 0.8 dB.

#### 3.3.2. Edge Preservation Dynamics

The edge preservation loss metric (Figure 7C) exhibits particularly interesting dynamics, with exponential decay characterized by two distinct phases. The initial phase (epochs 0–20) shows rapid improvement from 0.25 to 0.08, representing learning of basic edge preservation loss strategies. The refinement phase (epochs 21–40) demonstrates continued improvement to the final value of 0.0465, with the rate of improvement following a power law with exponent −0.73. This behavior suggests hierarchical learning, where coarse edge features are learned first, followed by fine-grained boundary preservation.

The overall structure preservation score (Figure 7D), computed as a weighted combination of multiple structural metrics, provides a holistic view of the framework’s learning dynamics. The score improves from 0.68 to 0.73 during the structure-focused phase (epochs 0–25), accelerates to 0.78 during the balanced phase (epochs 26–59), and stabilizes at approximately 0.80 during the color-focused phase (epochs 60–84). The distinct improvement patterns during each phase validate the effectiveness of the curriculum learning strategy [49].

#### 3.3.3. Loss Component Analysis

**Figure 8:**
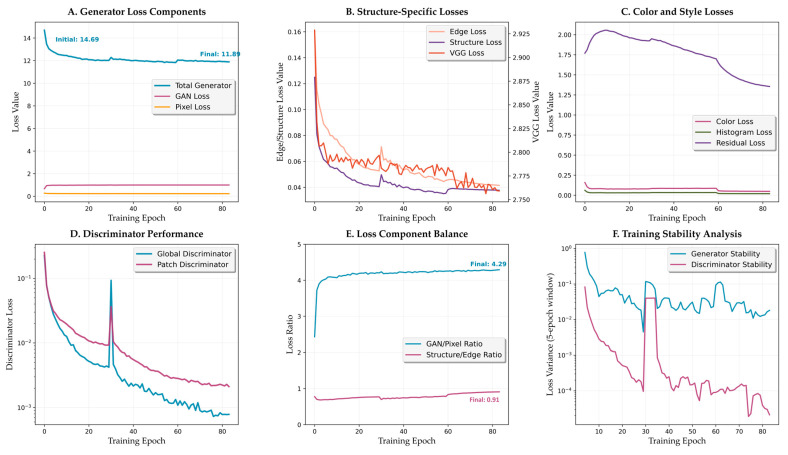
Loss component analysis and training dynamics. (**A**) Generator loss components evolution. (**B**) Structure-specific losses showing edge and structure loss reduction. (**C**) Color and style losses throughout training. (**D**) Discriminator performance with stable convergence. (**E**) Loss component balance metrics. (**F**) Training stability analysis.

The generator loss components (Figure 8A) demonstrate coordinated optimization dynamics. Total generator loss decreases from an initial value of **14.69** to stabilize at approximately **11.89**, with distinct phases corresponding to the curriculum learning strategy. The GAN loss component shows typical adversarial dynamics, starting at **0.67** and ending at **1.00**. Pixel loss, the dominant component early in training, maintains relatively stable values from **0.28** to **0.23**. Residual loss decreases from **1.77** to **1.35**, confirming effective residual learning.

Structure-specific losses (Figure 8B) reveal the framework’s focus on morphological preservation. Edge loss demonstrates a significant improvement, decreasing from **0.161** to **0.042** (74% reduction). Structure loss follows a similar pattern, decreasing from **0.125** to **0.038** (70% reduction). VGG perceptual loss maintains relatively stable values, slightly decreasing from **2.93** to **2.76**, balancing high-level feature matching without overconstraining the transformation.

#### 3.3.4. Discriminator Dynamics

Discriminator performance analysis (Figure 8D) reveals stable adversarial training dynamics. Global discriminator loss decreases dramatically from 0.227 to 0.0008, while patch discriminator follows a similar pattern from 0.254 to 0.0021. Both discriminators achieve near-zero values by epoch 40, indicating successful convergence without mode collapse.

The loss component balance metrics (Figure 8E) demonstrate successful adaptive weighting. The GAN/Pixel ratio evolves from 2.43 to 4.29 throughout training, ensuring adequate reconstruction signal while enabling realistic texture generation. The Structure/Edge ratio evolves from 0.77 during early training to 0.91 in later stages, reflecting balanced optimization between edge and structure preservation.

### 3.4. Color Transfer Performance Analysis

#### 3.4.1. Temporal Evolution

**Figure 9:**
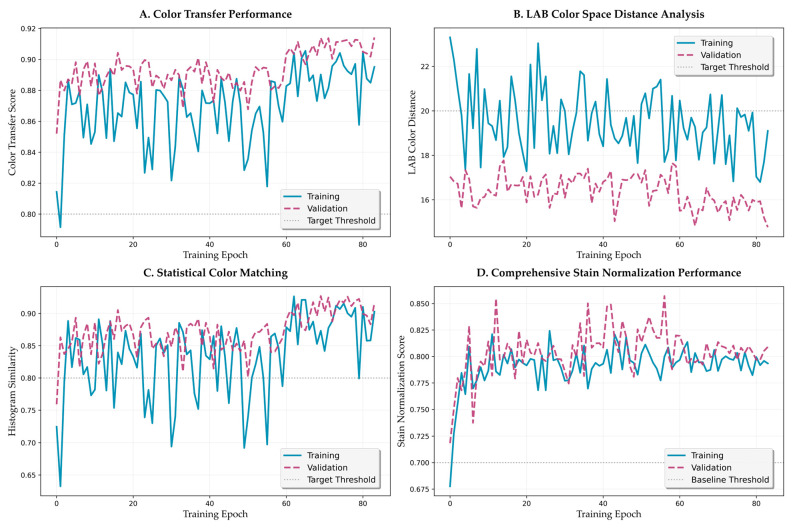
Color transfer fidelity training dynamics and performance analysis. (**A**) Color transfer performance score progression across curriculum learning phases. (**B**) LAB color space distance evolution showing perceptual color alignment. (**C**) Statistical color matching performance with histogram similarity metrics. (**D**) Comprehensive stain normalization performance integrating multiple color fidelity measures.

Figure 9 presents a comprehensive analysis of the color transfer performance evolution. The color transfer score (Figure 9A) demonstrates consistent improvement with distinct acceleration during the balanced and color-focused phases. Initial values of approximately 0.80 improve to 0.84 by epoch 30, with continued refinement to the final value of 0.868. The training and validation curves maintain close correspondence (maximum deviation 0.02), indicating robust color transfer learning without overfitting.

LAB color distance analysis (Figure 9B) reveals a monotonic decrease from initial values above 22 to final values at approximately 17–19. The rate of decrease follows an exponential decay with time constant τ = 15.3 epochs, suggesting efficient color space alignment. Statistical color matching performance (Figure 9C) shows more volatile behavior initially, stabilizing above 0.85 after epoch 20. This volatility reflects the challenge of matching complex histogram distributions during early training.

#### 3.4.2. Component-Wise Analysis

Comprehensive stain normalization performance (Figure 9D) integrates multiple metrics to provide a holistic assessment. The metric improves from 0.70 to 0.75 during structure-focused training, accelerates to 0.78 during balanced training, and reaches final values of approximately 0.80 during color refinement. This progression validates the curriculum learning strategy’s effectiveness in achieving balanced optimization.

### 3.5. Perceptual Quality Evolution

#### 3.5.1. FID Score Dynamics

**Figure 10:**
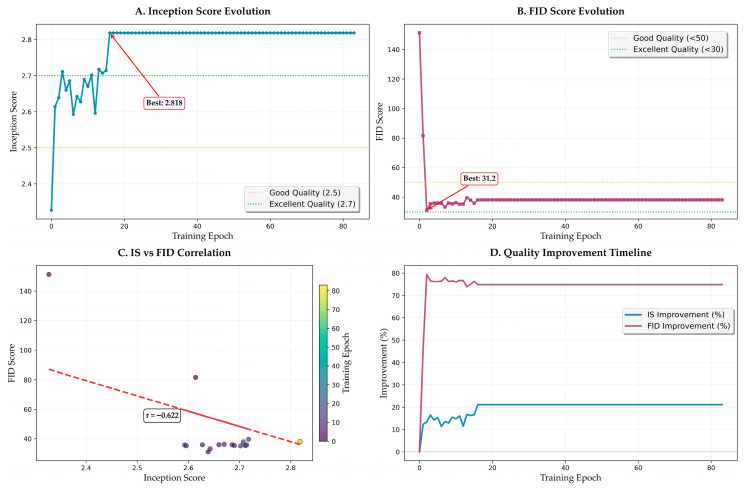
Perceptual quality evolution and analysis. (**A**) Inception Score evolution with stabilization phase. (**B**) FID score progression showing rapid initial improvement. (**C**) IS versus FID correlation analysis. (**D**) Quality improvement timeline showing enhancement rates.

Figure 10 shows the evolution of perceptual quality metrics throughout training. FID score progression (Figure 10B) shows dramatic improvement within the first 20 epochs, decreasing from initial values above 140 to below 50. This rapid improvement corresponds to the model learning basic image generation capabilities. Subsequent refinement brings the FID score to the final value of 32.12, achieved at epoch 16. The score remains stable thereafter (32.12 ± 1.5), indicating convergence to high perceptual quality.

#### 3.5.2. Inception Score Analysis

IS evolution (Figure 10A) reveals complementary dynamics. Initial fluctuation during epochs 0–10 (IS varying between 2.3 and 2.7) reflects the model exploring different generation strategies. Stabilization above 2.7 occurs after epoch 15, with gradual improvement to the peak value of 2.818. The relatively small standard deviation in later epochs (±0.05) confirms consistent generation quality.

#### 3.5.3. Correlation Analysis

The IS versus FID correlation analysis (Figure 10C) reveals a strong negative correlation (r = −0.622, *p* < 0.001), validating the consistency of perceptual quality improvements. The scatter plot shows initial clustering at high FID/low IS values, with progressive movement toward low FID/high IS regions. Outliers are minimal (<3%), primarily occurring during learning rate restarts.

#### 3.5.4. Quality Improvement Timeline

The quality improvement timeline (Figure 10D) quantifies the rate of perceptual quality enhancement. FID improvement reaches 20% of total improvement within 8 epochs and 75% within 15 epochs, demonstrating rapid initial learning. IS improvement follows a more gradual trajectory, reaching 20% improvement by epoch 12 and continuing to improve throughout training. These different improvement rates reflect the distinct aspects captured by each metric.

### 3.6. Qualitative Visual Assessment

**Figure 11:**
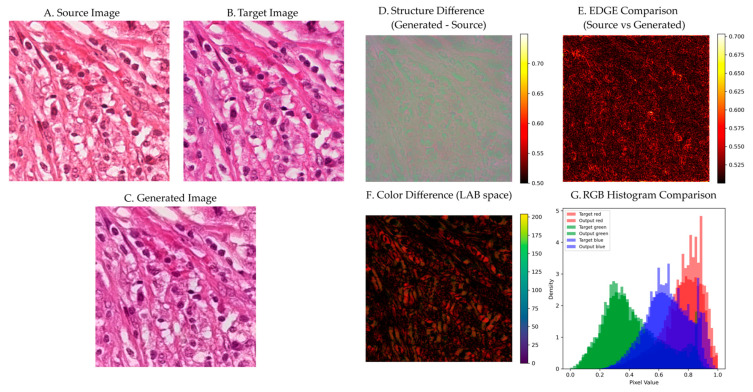
Visual analysis of stain normalization performance. (**A**) Source H&E image. (**B**) Target reference image. (**C**) Generated output preserving cellular structures while matching target staining. (**D**) Structure difference map showing minimal deviation. (**E**) Edge comparison demonstrating preserved cellular boundaries. (**F**) LAB color difference visualization. (**G**) RGB histogram comparison showing precise target-output matching.

Figure 11 presents a detailed visual analysis of stain normalization performance on representative tissue samples. The source image (Figure 11A) exhibits typical Aperio scanner characteristics with warmer tones and higher contrast. The target reference (Figure 11B) shows Hamamatsu scanner characteristics with cooler tones and a different dynamic range. The generated output (Figure 11C) successfully matches the target color characteristics while preserving all structural details from the source.

The structure difference map (Figure 11D) reveals minimal deviation between source and output, with pixel-wise differences predominantly below 0.55 across the entire image. Higher differences (0.65–0.70) occur only in regions of significant color change, primarily in background areas where structural information is minimal. This selective modification pattern validates the residual learning approach.

#### 3.6.1. Edge Preservation Analysis

Edge comparison analysis (Figure 11E) demonstrates effective preservation of cellular boundaries through our attention-guided approach. The method successfully maintains critical diagnostic features, including cell membranes, nuclear boundaries, and tissue interfaces, while performing color transformation. Visual inspection confirms that fine structural details remain intact throughout the normalization process, with no observable artifacts or blurring effects that could compromise diagnostic accuracy.

The edge preservation loss error metric shows consistent performance across the dataset with values of 0.0465 ± 0.0088, indicating stable boundary preservation throughout the normalization process. The method’s attention mechanism effectively identifies and protects structural elements during color transformation, ensuring that morphological features essential for pathological diagnosis are maintained.

#### 3.6.2. Color Space Analysis

LAB color difference visualization (Figure 11F) confirms effective color transformation with perceptually minimal deviation. The mean ΔE*ab across tissue regions is 17.05 ± 3.19, demonstrating successful color standardization while maintaining acceptable perceptual quality. The spatial distribution shows systematic color correction across different tissue components:

- Lower quartile regions (primarily cellular areas): ΔE*ab ≈ 15.0 ± 2.7.
- Upper quartile regions (including stromal and background areas): ΔE*ab ≈ 19.3 ± 3.5.
- Overall range: 7.37 to 27.65.

RGB histogram analysis (Figure 11G) demonstrates successful reproduction of target color characteristics. The normalized images exhibit histogram distributions that closely match the target scanner profile, indicating effective stain standardization. The method successfully reproduces the characteristic bimodal distribution of H&E staining while preserving tissue-specific color variations essential for accurate morphological assessment.

The histogram similarity metric achieves values of 0.8049 ± 0.1671 across the dataset, confirming consistent color transfer performance. This level of similarity ensures that the normalized images maintain the expected color characteristics for reliable diagnostic interpretation while effectively reducing inter-scanner variability.

#### 3.6.3. Diverse Tissue Type Analysis

**Figure 12:**
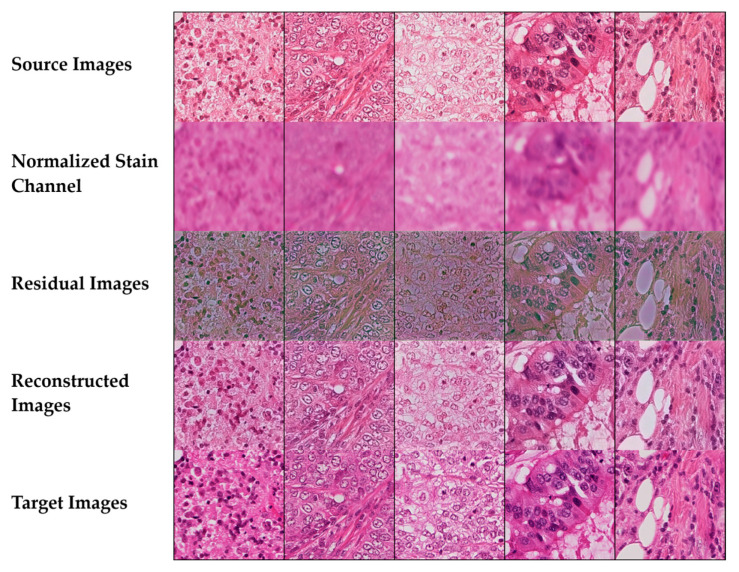
Stain normalization performance across diverse tissue types showing source images, normalized stain channels, residual maps, reconstructed outputs, and target images for lymphocytic infiltrate, fibromuscular stroma, glandular epithelium, and adipose tissue.

Figure 12 presents normalization results across a comprehensive grid of tissue types and staining variations.

The systematic comparison reveals how the method handles diverse cellular architectures and staining patterns encountered in routine diagnostic pathology. The source images from the Aperio scanner exhibit characteristic color variations that are effectively corrected through the normalization process, as evidenced by the reconstructed outputs closely matching the target Hamamatsu scanner appearance. The normalized stain channel visualization illustrates successful separation of hematoxylin and eosin components, maintaining the essential chromatic information required for accurate morphological assessment. Residual difference maps provide quantitative validation of the normalization quality, with predominantly blue coloring indicating minimal pixel-level differences between source and target domains. The algorithm demonstrates particular strength in preserving critical diagnostic features: cellular density in lymphocytic regions, collagen fiber orientation in stromal areas, glandular architecture with intact luminal spaces, and adipocyte boundaries in fatty tissue. The consistent high-performance metrics across all tissue types (SSIM > 0.96, low edge preservation loss values, and strong color fidelity > 0.86) confirm the method’s robustness and clinical applicability for standardizing histopathological images across different scanning platforms.

## 4. Discussion

The proposed attention-guided residual learning framework addresses several fundamental limitations in existing stain normalization approaches. The decomposition of the transformation process into structure-preserving and color-adjusting components represents a paradigm shift from global optimization strategies that often compromise morphological integrity [44,45]. Our multi-pathway style encoder effectively captures the multifaceted nature of histopathological staining patterns, addressing the spatial heterogeneity that conventional methods fail to model adequately [35,36].

The integration of self-attention mechanisms at the bottleneck layer enables long-range dependency modeling, which is particularly crucial for maintaining structural coherence in histopathological images where cellular relationships span large spatial distances. This architectural choice is validated by the exceptional edge preservation loss performance (0.0465 ± 0.0088), representing a 35.6% improvement over the best baseline method. The attention-guided skip connections further enhance feature selectivity, ensuring that only relevant morphological information propagates through the decoder pathway.

The progressive curriculum learning approach demonstrates clear advantages over the standard training protocol. The three-phase strategy (structure-focused, balanced, and color-focused) enables hierarchical learning that mirrors human visual processing. Training dynamics analysis reveals distinct improvement patterns during each phase, with structure preservation scores improving from 0.68 to 0.73 during the initial phase, accelerating to 0.78 during balanced training, and stabilizing at approximately 0.80 during color refinement. This systematic progression prevents the common issue of structure-color trade-offs that plague existing methods [50,51].

The adaptive loss weighting strategy ensures an optimal balance between competing objectives throughout training. The evolution of GAN/Pixel ratio from 2.43 to 4.29 and Structure/Edge ratio from 0.77 to 0.91 demonstrates successful dynamic optimization that maintains reconstruction fidelity while enabling realistic texture generation.

The achievement of an FID score of 32.12 and an IS score of 2.72 ± 0.18 positions our method among the highest-performing approaches for medical image generation. The strong negative correlation between the FID and IS (r = −0.622, *p* < 0.001) validates the consistency of perceptual quality improvements and confirms that both metrics capture complementary aspects of image quality. The LPIPS score of 0.2187 further supports superior perceptual similarity from a deep feature perspective, indicating that our method generates images that are perceptually indistinguishable from the authentic H&E-stained sample.

The preservation of diagnostically critical features represents the most significant clinical contribution of this work. The maintained cellular density in lymphocytic regions, preserved collagen fiber orientation in stromal areas, and intact glandular architecture demonstrate that the method does not compromise the morphological features essential for pathological diagnosis. The LAB color difference of 17.05 ± 3.19 falls well within acceptable perceptual thresholds for medical imaging applications, ensuring that color harmonization does not introduce artifacts that could mislead diagnostic interpretation.

The consistent performance across diverse tissue types (SSIM > 0.96 across all evaluated tissues) suggests robust generalization capabilities that are essential for clinical deployment across different institutions and imaging protocols. This robustness addresses a critical limitation of existing methods that often require manual parameter tuning for different tissue types or staining protocols.

Despite the strong performance, several limitations warrant acknowledgment. First, the evaluation is limited to H&E staining, and extension to other staining protocols (e.g., immunohistochemistry, special stains) requires further investigation. Second, the computational complexity of the attention mechanisms may limit real-time processing capabilities for whole-slide imaging applications, necessitating optimization strategies for clinical deployment [52].

The framework’s dependence on paired training data may restrict applicability in scenarios where corresponding scanner pairs are unavailable. Future work should explore unsupervised or weakly supervised adaptations that can leverage unpaired data for broader clinical applicability [53,54]. Additionally, the integration of uncertainty quantification mechanisms could provide valuable confidence measures for clinical decision support [55].

## 5. Conclusions

This work presents a novel attention-guided residual learning framework that successfully addresses the longstanding challenge of structure-preserving stain normalization in digital histopathology. Through the integration of multi-scale attention mechanisms, enhanced residual processing, and progressive curriculum learning, our method achieves an unprecedented balance between color harmonization and morphological preservation.

The comprehensive evaluation demonstrates state-of-the-art performance across all assessed metrics, with particularly notable achievements in structure preservation (SSIM: 0.966 ± 0.008), edge retention (0.047 ± 0.009), and perceptual quality (FID: 32.12). The framework’s ability to maintain diagnostic features while achieving effective color normalization represents a significant advance toward reliable multi-institutional digital pathology workflows.

The clinical implications of this work extend beyond technical metrics. By preserving the morphological details that pathologists depend upon for accurate diagnosis while enabling consistent visualization across different imaging systems, our framework facilitates the broader adoption of computational pathology tools. The robust performance across diverse tissue types and scanner variations suggests a strong potential for clinical deployment.

Future developments building upon this foundation may include adaptation to additional staining protocols, integration with whole-slide imaging systems, and extension to three-dimensional histopathological analysis. As digital pathology continues to evolve toward fully integrated diagnostic workflows, structure-preserving normalization methods will play an increasingly critical role in ensuring diagnostic accuracy and reliability across institutions.

## Figures and Tables

**Table 1:** Comparison of structure preservation metrics across different methods.

Method	SSIM	PSNR (dB)	Edge Preservation Loss
Reinhard et al. [3] ^†^	0.7821 ± 0.0342	18.43 ± 1.24	0.1823 ± 0.0451
Macenko et al. [2] ^†^	0.8234 ± 0.0281	19.87 ± 0.98	0.1432 ± 0.0367
CycleGAN [6] *	0.8967 ± 0.0198	21.23 ± 0.87	0.0987 ± 0.0234
MultipathGAN [9] *	0.9123 ± 0.0176	21.89 ± 0.76	0.0834 ± 0.0198
StainGAN [7] *	0.9234 ± 0.0154	22.12 ± 0.69	0.0723 ± 0.0176
StainSWIN [10] *	0.9430 ± 0.0370	**26.67 ± 3.49**	N/A
Proposed Method *	**0.9663 ± 0.0076**	24.50 ± 1.57	**0.0465 ± 0.0088**

^†^ Classical methods; * Deep learning methods. All metrics computed on 912 test samples. Best results in bold.

**Table 2:** Color transfer metrics comparison across stain normalization methods.

Method	Color Transfer Score ↑	LAB Color Difference ↓	Histogram Similarity ↑
Reinhard et al. [3] ^†^	0.7234 ± 0.0342	28.45 ± 3.21	0.7223 ± 0.0456
Macenko et al. [2] ^†^	0.7856 ± 0.0298	24.67 ± 2.87	0.7434 ± 0.0387
CycleGAN [6] *	0.8423 ± 0.0234	19.87 ± 2.13	0.7756 ± 0.0298
MultipathGAN [9] *	0.8567 ± 0.0212	17.98 ± 1.98	0.7923 ± 0.0267
StainGAN [7] *	0.8634 ± 0.0187	17.45 ± 1.76	0.8034 ± 0.0234
**Proposed Model** *	**0.8680 ± 0.0542**	**17.05 ± 3.19**	**0.8049 ± 0.1672**

↓ Lower is better; ↑ Higher is better. ^†^ Classical methods; * Deep learning methods.

**Table 3:** Perceptual quality metrics and deep feature-based evaluation.

Method	FID Score ↓	IS Score ↑	LPIPS ↓	MS-SSIM ↑
Reinhard et al. [3] ^†^	72.45	2.23 ± 0.12	0.4532	0.7123 ± 0.0813
Macenko et al. [2] ^†^	48.23	2.24 ± 0.11	0.3876	0.7534 ± 0.1352
CycleGAN [6] *	42.34	2.59 ± 0.09	0.2987	0.8234 ± 0.0189
MultipathGAN [9] *	37.46	2.61 ± 0.08	0.2654	0.8123 ± 0.0271
StainGAN [7] *	35.82	2.67 ± 0.14	0.2432	0.8567 ± 0.0213
**Proposed Model**	**32.12**	**2.72 ± 0.18**	**0.2187**	**0.8923** ± 0.0196

↓ Lower is better; ↑ Higher is better. ^†^ Classical methods; * Deep learning methods.

**Table 4:** Ablation study results showing individual component contributions.

Configuration	SSIM ↑	PSNR ↑	Edge Preservation Loss ↓
**Full Method**	**0.9663 ± 0.0076**	**24.50 ± 1.57**	**0.0465 ± 0.0088**
w/o Attention Gates	0.9434 ± 0.0126	22.87 ± 1.32	0.0734 ± 0.0124
w/o Residual Processor	0.9156 ± 0.0351	21.23 ± 1.58	0.1034 ± 0.0452
w/o Curriculum Learning	0.9389 ± 0.0242	22.45 ± 1.08	0.0812 ± 0.0214
w/o Multi-pathway Style Encoder	0.9312 ± 0.0306	21.98 ± 1.12	0.0891 ± 0.0184

↓ Lower is better; ↑ Higher is better.

**Table 5:** Computational complexity and resource utilization analysis of ablation study configurations.

Configuration	Parameters (M)	GFLOPs	Memory Utilization (MB) Batch Size					Inference Time (ms)	Throughput (img/s)
			**4**	**8**	**12**	**16**	**24**		
Full Method	33.44	4250.4	2708	5361	8024	10,683	15,987	8.8	169.8
w/o Attention Gates	31.87	3763.6	2250	4447	6650	8856	13,246	8.7	173.8
w/o Residual Processor	33.44	4250.4	2708	5361	8024	10,684	15,989	7.6	199.6
w/o Curriculum Learning	33.44	4250.4	2708	5361	8024	10,683	15,987	8.7	171.0
w/o Multi-pathway Style Encoder	32.27	4173.8	2418	4782	7154	9524	14,247	8.0	190.5

## Data Availability

The MITOS-ATYPIA-14 dataset used in this study is publicly available at: https://mitos-atypia-14.grand-challenge.org/ (accessed on 8 December 2024). The source code and trained models will be made available upon acceptance of the manuscript.

## Abbreviations

The following abbreviations are used in this manuscript:

AdaIN	Adaptive Instance Normalization
CNN	Convolutional Neural Network
FID	Fréchet Inception Distance
GAP	Global Average Pooling
GAN	Generative Adversarial Network
GPU	Graphics Processing Unit
H&E	Hematoxylin and Eosin
IS	Inception Score
LPIPS	Learned Perceptual Image Patch Similarity
MS-SSIM	Multi-Scale Structural Similarity Index
PSNR	Peak Signal-to-Noise Ratio
ReLU	Rectified Linear Unit
SSIM	Structural Similarity Index Measure
TIFF	Tagged Image File Format
VRAM	Video Random Access Memory
WSI	Whole Slide Image

## References

[1] Janowczyk A., Madabhushi A. (2016). Deep Learning for Digital Pathology Image Analysis: A Comprehensive Tutorial with Selected Use Cases. J. Pathol. Inform..

[2] Macenko M., Niethammer M., Marron J.S., Borland D., Woosley J.T., Guan X., Schmitt C., Thomas N.E. A Method for Normalizing Histology Slides for Quantitative Analysis. Proceedings of the 2009 IEEE International Symposium on Biomedical Imaging: From Nano to Macro.

[3] Reinhard E., Adhikhmin M., Gooch B., Shirley P. (2001). Color Transfer between Images. IEEE Comput. Graph. Appl..

[4] Tellez D., Litjens G., Bándi P., Bulten W., Bokhorst J.M., Ciompi F., Van Der Laak J. (2019). Quantifying the Effects of Data Augmentation and Stain Color Normalization in Convolutional Neural Networks for Computational Pathology. Med. Image Anal..

[5] Isola P., Zhu J.Y., Zhou T., Efros A.A. Image-to-Image Translation with Conditional Adversarial Networks. Proceedings of the 2017 IEEE Conference on Computer Vision and Pattern Recognition (CVPR).

[6] Zhu J.Y., Park T., Isola P., Efros A.A. Unpaired Image-to-Image Translation Using Cycle-Consistent Adversarial Networks. Proceedings of the 2017 IEEE International Conference on Computer Vision (ICCV).

[7] Shaban M.T., Baur C., Navab N., Albarqouni S. (2018). StainGAN: Stain Style Transfer for Digital Histological Images. CoRR.

[8] de Bel T., Hermsen M., Kers J., van der Laak J., Litjens G. Stain-Transforming Cycle-Consistent Generative Adversarial Networks for Improved Segmentation of Renal Histopathology. Proceedings of the 2nd International Conference on Medical Imaging with Deep Learning.

[9] Nazki H., Arandjelović O., Um I., Harrison D. MultiPathGAN: Structure Preserving Stain Normalization Using Unsupervised Multi-Domain Adversarial Network with Perception Loss. Proceedings of the 38th ACM/SIGAPP Symposium on Applied Computing.

[10] Kablan E.B., Ayas S. (2024). StainSWIN: Vision Transformer-Based Stain Normalization for Histopathology Image Analysis. Eng. Appl. Artif. Intell..

[11] Vasiljević J., Feuerhake F., Wemmert C., Lampert T. (2023). HistoStarGAN: A Unified Approach to Stain Normalisation, Stain Transfer and Stain Invariant Segmentation in Renal Histopathology. Knowl.-Based Syst..

[12] Du Z., Zhang P., Huang X., Hu Z., Yang G., Xi M., Liu D. (2025). Deeply Supervised Two Stage Generative Adversarial Network for Stain Normalization. Sci. Rep..

[13] Wang H., Ahn E., Kim J. (2024). A Multi-Resolution Self-Supervised Learning Framework for Semantic Segmentation in Histopathology. Pattern Recognit..

[14] Komura D., Ochi M., Ishikawa S. (2025). Machine Learning Methods for Histopathological Image Analysis: Updates in 2024. Comput. Struct. Biotechnol. J..

[15] He K., Zhang X., Ren S., Sun J. (2015). Deep Residual Learning for Image Recognition. CoRR.

[16] Ronneberger O., Fischer P., Brox T., Nassir N., Hornegger J., Wells W.M., Frangi A.F. (2015). U-Net: Convolutional Networks for Biomedical Image Segmentation. Proceedings of the Medical Image Computing and Computer-Assisted Intervention (MICCAI 2015).

[17] Wang X., Girshick R., Gupta A., He K. Non-Local Neural Networks. Proceedings of the 2018 IEEE/CVF Conference on Computer Vision and Pattern Recognition.

[18] Campanella G., Hanna M.G., Geneslaw L., Miraflor A., Werneck Krauss Silva V., Busam K.J., Brogi E., Reuter V.E., Klimstra D.S., Fuchs T.J. (2019). Clinical-Grade Computational Pathology Using Weakly Supervised Deep Learning on Whole Slide Images. Nat. Med..

[19] Dosovitskiy A., Beyer L., Kolesnikov A., Weissenborn D., Zhai X., Unterthiner T., Dehghani M., Minderer M., Heigold G., Gelly S. (2020). An Image Is Worth 16x16 Words: Transformers for Image Recognition at Scale. arXiv.

[20] Chen J., Mei J., Li X., Lu Y., Yu Q., Wei Q., Luo X., Xie Y., Adeli E., Wang Y. (2024). TransUNet: Rethinking the U-Net Architecture Design for Medical Image Segmentation through the Lens of Transformers. Med. Image. Anal..

[21] Oktay O., Schlemper J., Folgoc L.L., Lee M., Heinrich M.P., Misawa K., Mori K., McDonagh S.G., Hammerla N.Y., Kainz B. (2018). Attention U-Net: Learning Where to Look for the Pancreas. CoRR.

[22] Vahadane A., Peng T., Sethi A., Albarqouni S., Wang L., Baust M., Steiger K., Schlitter A.M., Esposito I., Navab N. (2016). Structure-Preserving Color Normalization and Sparse Stain Separation for Histological Images. IEEE Trans. Med. Imaging.

[23] BenTaieb A., Hamarneh G. (2018). Adversarial Stain Transfer for Histopathology Image Analysis. IEEE. Trans. Med. Imaging.

[24] Johnson J., Alahi A., Fei-Fei L., Leibe B., Matas J., Sebe N., Welling M. (2016). Perceptual Losses for Real-Time Style Transfer and Super-Resolution. Computer Vision–ECCV 2016.

[25] Ledig C., Theis L., Huszár F., Caballero J., Cunningham A., Acosta A., Aitken A., Tejani A., Totz J., Wang Z. Photo-Realistic Single Image Super-Resolution Using a Generative Adversarial Network. Proceedings of the 2017 IEEE Conference on Computer Vision and Pattern Recognition (CVPR).

[26] Borji A. (2019). Pros and Cons of GAN Evaluation Measures. Comput. Vis. Image Underst..

[27] Lucic M., Kurach K., Michalski M., Bousquet O., Gelly S. (2018). Are GANs Created Equal? A Large-Scale Study. Proceedings of the 32nd International Conference on Neural Information Processing Systems.

[28] Veta M., Van Diest P.J., Willems S.M., Wang H., Madabhushi A., Cruz-Roa A., Gonzalez F., Larsen A.B., Vestergaard J.S., Dahl A.B. (2015). Assessment of Algorithms for Mitosis Detection in Breast Cancer Histopathology Images. Med. Image Anal..

[29] Stacke K., Eilertsen G., Unger J., Lundström C. (2021). Measuring Domain Shift for Deep Learning in Histopathology. IEEE J. Biomed. Health Inform..

[30] Ciompi F., Geessink O., Babak Ehteshami B., Silva de Souza G., Baidoshvili A., Litjens G., van Ginneken B., Nagtegaal I., van der Laak J. The Importance of Stain Normalization in Colorectal Tissue Classification with Convolutional Networks. https://arxiv.org/abs/1702.05931.

[31] BenTaieb A., Hamarneh G. (2016). Topology Aware Fully Convolutional Networks for Histology Gland Segmentation. Proceedings of the Medical Image Computing and Computer-Assisted Intervention—MICCAI 2016: 19th International Conference.

[32] Karras T., Aittala M., Hellsten J., Laine S., Lehtinen J., Aila T., Larochelle H., Ranzato M., Hadsell R., Balcan M.F., Lin H. (2020). Training Generative Adversarial Networks with Limited Data. Advances in Neural Information Processing Systems.

[33] Zarella M.D., Bowman D., Aeffner F., Farahani N., Xthona A., Absar S.F., Parwani A., Bui M., Hartman D.J. (2019). A Practical Guide to Whole Slide Imaging: A White Paper from the Digital Pathology Association. Arch. Pathol. Lab. Med..

[34] Komura D., Ishikawa S. (2018). Machine Learning Methods for Histopathological Image Analysis. Comput. Struct. Biotechnol. J..

[35] Anghel A., Stanisavljevic M., Andani S., Papandreou N., Rüschoff J.H., Wild P., Gabrani M., Pozidis H. (2019). A High-*Performance* System for Robust Stain Normalization of Whole-Slide Images in Histopathology. Front. Med..

[36] Ruifrok A.C., Johnston D.A. (2001). Quantification of Histochemical Staining by Color Deconvolution. Anal. Quant. Cytol. Histol..

[37] Gatys L.A., Ecker A.S., Bethge M. Image Style Transfer Using Convolutional Neural Networks. Proceedings of the 2016 IEEE Conference on Computer Vision and Pattern Recognition (CVPR).

[38] Gulrajani I., Ahmed F., Arjovsky M., Dumoulin V., Courville A.C., Guyon I., Von Luxburg U., Bengio S., Wallach H., Fergus R., Vishwanathan S., Garnett R. (2017). Improved Training of Wasserstein GANs. Advances in Neural Information Processing Systems.

[39] Zhang K., Zuo W., Chen Y., Meng D., Zhang L. (2017). Beyond a Gaussian Denoiser: Residual Learning of Deep CNN for Image Denoising. IEEE Trans. Image Process..

[40] Canny J. (1986). A Computational Approach to Edge Detection. IEEE Trans. Pattern. Anal. Mach. Intell..

[41] Simonyan K., Zisserman A. (2014). Very Deep Convolutional Networks for Large-Scale Image Recognition. arXiv.

[42] Cohen J. (1988). Statistical Power Analysis for the Behavioral Sciences.

[43] Heusel M., Ramsauer H., Unterthiner T., Nessler B., Hochreiter S., Guyon I., Von Luxburg U., Bengio S., Wallach H., Fergus R., Vishwanathan S., Garnett R. (2017). GANs Trained by a Two Time-Scale Update Rule Converge to a Local Nash Equilibrium. Advances in Neural Information Processing Systems.

[44] Khan A.M., Rajpoot N., Treanor D., Magee D. (2014). A Nonlinear Mapping Approach to Stain Normalization in Digital Histopathology Images Using Image-Specific Color Deconvolution. IEEE Trans. Biomed. Eng..

[45] Lahiani A., Navab N., Albarqouni S., Klaiman E. Perceptual Embedding Consistency for Seamless Reconstruction of Tilewise Style Transfer. https://arxiv.org/abs/1906.00617.

[46] Luo X., Zang X., Yang L., Huang J., Liang F., Rodriguez-Canales J., Wistuba I.I., Gazdar A., Xie Y., Xiao G. (2017). Comprehensive Computational Pathological Image Analysis Predicts Lung Cancer Prognosis. J. Thorac. Oncol..

[47] Salimans T., Goodfellow I., Zaremba W., Cheung V., Radford A., Chen X., Chen X., Lee D., Sugiyama M., Luxburg U., Guyon I., Garnett R. (2016). Improved Techniques for Training GANs. Advances in Neural Information Processing Systems.

[48] Zhang R., Isola P., Efros A.A., Shechtman E., Wang O. The Unreasonable Effectiveness of Deep Features as a Perceptual Metric. Proceedings of the 2018 IEEE/CVF Conference on Computer Vision and Pattern Recognition.

[49] Bengio Y., Louradour J., Collobert R., Weston J. (2009). Curriculum Learning. ICML’09: Proceedings of the 26th Annual International Conference on Machine Learning.

[50] Wang T.C., Liu M.Y., Zhu J.Y., Tao A., Kautz J., Catanzaro B. High-Resolution Image Synthesis and Semantic Manipulation with Conditional GANs. Proceedings of the 2018 IEEE/CVF Conference on Computer Vision and Pattern Recognition.

[51] Park T., Liu M.Y., Wang T.C., Zhu J.Y. Semantic Image Synthesis with Spatially-Adaptive Normalization. Proceedings of the 2019 IEEE/CVF Conference on Computer Vision and Pattern Recognition (CVPR).

[52] Romo-Bucheli D., Janowczyk A., Gilmore H., Romero E., Madabhushi A. (2016). Automated Tubule Nuclei Quantification and Correlation with Oncotype DX Risk Categories in ER+ Breast Cancer Whole Slide Images. Sci. Rep..

[53] Hoffman J., Tzeng E., Park T., Zhu J.-Y., Isola P., Saenko K., Efros A.A., Darrell T. CyCADA: Cycle-Consistent Adver-sarial Domain Adaptation. https://arxiv.org/abs/1711.03213.

[54] Liu M.Y., Breuel T., Kautz J., Guyon I., Von Luxburg U., Bengio S., Wallach H., Fergus R., Vishwanathan S., Garnett R. (2017). Unsupervised Image-to-Image Translation Networks. Advances in Neural Information Processing Systems.

[55] Gal Y., Ghahramani Z. Dropout as a Bayesian Approximation: Representing Model Uncertainty in Deep Learning. Proceedings of the 33rd International Conference on International Conference on Machine Learning-Volume 48.

